# Tuboovarian Abscesses: Is Size Associated with Duration of Hospitalization & Complications?

**DOI:** 10.1155/2010/847041

**Published:** 2010-05-24

**Authors:** Jason DeWitt, Angela Reining, Jenifer E. Allsworth, Jeffrey F. Peipert

**Affiliations:** Division of Clinical Research, Department of Obstetrics and Gynecology, Washington University School of Medicine in St. Louis, St. Louis, MO 63110, USA

## Abstract

*Objective*. To evaluate the association between abscess size and duration of hospitalization and need for surgical intervention. *Methods*. We collected data from patients admitted with ICD-9 codes 614.9 (PID) and 614.2 (TOA) from January 1, 1999—December 31, 2005. We abstracted data regarding demographics, diagnostic testing/laboratory testing, imaging, treatment, and clinical course. Two abscess groups were created: ≤8 cm or >8 cm. Descriptive statistics were calculated, and duration of hospitalization and surgical intervention for women with large abscesses were compared to women with smaller collections. *Results*. Of the 373 charts reviewed, 135 had a TOA and 31% required management with drainage and/or surgery. The average abscess size for those treated successfully with conservative management was 6.3 cm versus those requiring drainage and/or surgery (7.7 cm, *P* = .02). Every 1 cm increase in abscess size as associated with an increase in hospitalization by 0.4 days (*P* = .001). Abscesses greater than 8 cm were associated with an increased risk of complications (*P* < .01). *Conclusions*. Larger tubo-ovarian abscesses are associated with an increased duration of hospitalization and more complications including an increased need for drainage or surgery. Additional research to determine the most efficacious antibiotic regimen management strategy is needed.

## 1. Introduction

Tuboovarian abscess (TOA), typically the end result of acute pelvic inflammatory disease (PID), is a condition characterized by a walled-off inflammatory mass in the pelvis. One-third to one-half of patients diagnosed with a TOA acknowledge a history of PID [1, 2]. PID and TOAs are polymicrobial infections of anaerobic and aerobic bacteria. While *Neisseria gonorrhoeae* and *Chlamydia trachomatis* are thought to facilitate the infection, they are rarely recovered from an abscess [3, 4]. The most commonly isolated organisms from TOAs are *Escherichia coli* and Bacteroides species [5]. 

TOA is a complication of PID in 15% of cases, and 33% of patients with PID requiring admission have a TOA [5]. Mortality associated with TOA has decreased dramatically over the last 50 years. However, the morbidity associated with TOA remains significant with complications including infertility, ectopic pregnancy, chronic pelvic pain, pelvic thrombophlebitis, and ovarian vein thrombosis [6]. 

While the majority of TOA respond to antibiotic therapy, in approximately 25% of cases surgery or drainage is indicated [7]. There is some evidence that TOA size is associated with need for intervention. Reed et al. in 1991 showed that 35% of abscesses 7 to 9 cm required surgery and nearly 60% of abscesses ≥10 cm required surgery [8]. The objective of our study was to assess the association of TOA size and need for prolonged hospitalization and surgical intervention. Our hypothesis was that TOA size would be associated with prolonged hospitalization and increased need for surgery or drainage. 

## 2. Methods

After obtaining approval from the Washington University in St. Louis Human Research Protection Office, we performed a retrospective review of women diagnosed with TOA admitted to Barnes-Jewish Hospital in Saint Louis, Missouri, from January 1, 1999 until December 31, 2005. Potential subjects were selected by the following ICD-9 codes: PID (614.9) and TOA (614.2). Three hundred seventy-three charts were then reviewed to confirm eligibility. Inclusion criteria included (1) presence of an inflammatory mass in the pelvis, (2) age greater than 17 years, and (3) admission to Barnes-Jewish Hospital. We excluded charts of women who did not have an inflammatory mass identified. Data were abstracted for demographics, diagnostic testing, imaging, treatment, and clinical course. 

Imaging was obtained in almost all cases and included ultrasound, CT, or MRI. In just over half of cases more than one imaging modality was used. One abscess that was not imaged preoperatively was identified at laparotomy. Fever was defined as a temperature greater than 38.2°C. Complications were defined as factors that could result in prolonged hospitalization; specifically, complications included intensive care unit (ICU) admission, bowel or bladder injury, blood transfusion, blood loss >1000 mL, sepsis or bacteremia, ileus, readmission, and death. Data were abstracted by three trained abstractors. 

We created two abscess groups based on maximal diameter of the abscess: ≤8 cm and >8 cm. If bilateral abscesses were noted, we used the larger of the two to quantify the size of the abscess. Women with large and small abscesses were compared by duration of hospitalization, surgical intervention, and complication rate. The initial antibiotic regimens were categorized to include at least (1) cefoxitin and doxycycline, (2) ciprofloxacin and metronidazole, (3) gentamicin and clindamycin, and (4) others.

Descriptive statistics were calculated using mean, standard deviation, and percentages. Comparisons and associations were made using Chi-square or Fisher's exact test for categorical variables. *T*-tests were used for continuous variables; nonparametric tests were used for data that were not normally distributed. Estimates of relative risk were estimated using Poisson regression with robust error variance. This approach results in an unbiased estimate when a binary outcome is common (>10%) [9]. The power calculations with an *α* error of 0.05 and *β* error of 0.20 found that the number of TOAs needed to evaluate was 121 assuming that 15% of cases would have TOA < 8 cm and 30% would have TOA > 8 cm. To achieve our target of 121 cases of TOA, we estimated that 370 charts would need to be reviewed given the frequent misdiagnosis of PID. Statistical analyses were conducted using SAS v. 9.2 (SAS Corporation, Cary, NC).

## 3. Results

Of 373 charts reviewed, 135 patients were identified as having a TOA while 218 patients were excluded based on the lack of presence of an abscess. Demographic and reproductive characteristics are provided in Table 1. The average age was 35.2 years (SD 10.0), and the mean gravidity and parity were 1.96 (SD 1.76) and 1.52 (SD 1.64), respectively. More than two-thirds were black, and 10% gave a history of having had a bilateral tubal ligation. 

Clinical characteristics of the study population are noted in Table 2. The most common reason for presentation was pain (94%) with a median duration of 3 days. Only 27% of patients had a fever on admission. The mean white blood count (WBC) on admission was 14.2 (SD 5.9) with 27% having WBC less than 10,000. Few subjects had positive tests for *N. gonorrhoeae* (12%) and *C. trachomatis* (14%). Imaging was obtained in most cases with patients frequently having either a CT (79%) or an ultrasound (70%). MRI was used in only 3 cases (2%). In the only case in which imaging was not obtained, the TOA was diagnosed during her cesarean section and bilateral tubal ligation and confirmed on pathology. Abscess size ranged from 1.5 cm to 16 cm with a mean of 6.7 cm. Most (51%) abscesses were between 5 and 8 cm. Two-thirds of abscesses were unilateral. Of patients with bilateral abscesses, the maximal diameter of the smaller abscess was 8 cm.

Comparison was made between those patients with abscesses ≤8 cm or >8 cm (Table 3). Of the 135 patients with a TOA, 112 (83%) had an abscess ≤8 cm and 23 (17%) had an abscess >8 cm. Patients with larger abscesses averaged longer duration of hospitalization, more complications, longer duration febrile, and an increased need for surgery or drainage when compared with those patients with smaller abscesses. Every 1 cm increase in abscess size is associated with an increase in hospitalization by 0.4 days (*P* =  .001). The mean abscess size for those requiring drainage or surgery was 7.7 cm versus 6.3 cm (*P* =  .02) for those with successful conservative management. Among the larger abscess group, 7 hysterectomies (30%) were performed with 8 (35%) patients receiving a simple adnexectomy. Patients with abscesses ≤8 cm had fewer complications (9%) than did patients with abscesses >8 cm (35%, *P* <  .01). Patients often had more than one complication; 18 patients developed 35 complications. The most common complication was blood transfusion which occurred in 14 patients (10%). There were no deaths. Patients with larger abscesses were febrile for longer versus those with smaller abscesses (1.35 versus 1.10 days, *P* =  .65). 

Patients were then evaluated by initial antibiotic regimen (Table 4). Fifty-seven patients were initially placed on regimens containing cefoxitin and doxycycline, and of these, 91% were successfully managed conservatively. Patients placed initially on regimens including cefoxitin and doxycycline were less likely to require surgery or drainage. This is in contrast to other antibiotic regimens which had approximate equal need for surgery or drainage in the smaller and larger abscess groups. A regression model was used to assess the need for surgical intervention or drainage controlling for other confounders. The relative risk for cefoxitin/doxycycline regimen was 0.21 (95%, CI 0.09–0.48) adjusting for age and total number of antibiotics. This finding persisted when fever, abscess >8 cm, and days hospitalized were included in the model. 

## 4. Discussion

We found that TOA size is associated with important outcomes including more complications and longer duration of hospitalization as well as an increased need for surgery or drainage when compared with those patients with smaller abscesses. Reed et al. also found that increasing abscess size is associated with increasing need for operative management [8]. We found a 43% failure rate for abscesses >8 cm while Reed showed a 35% failure rate for abscesses 7 to 9 cm and nearly 60% failure rate for abscesses ≥10 cm. In contrast, Gjelland et al. noted that treatment success was not affected by the size of the abscess or the presence of bilateral abscesses in a sample restricted to abscesses with maximal diameter of at least 3 cm. The authors of this study recommended transvaginal drainage as a first-line procedure [10].

Originally, treatment of TOA was thought to require bilateral oophorectomy and hysterectomy. Medical management with broad spectrum antibiotics is now generally considered as the initial management for unruptured TOAs [11]. However, optimal treatment of TOA remains unclear. The 2006 Center for Disease Control and Prevention Sexually Transmitted Diseases Treatment Guidelines recommends inpatient intravenous antibiotics for at least 24 hours. No specific inpatient antibiotic regimen is suggested. Upon discontinuation of parenteral therapy, the CDC recommends that clindamycin or metronidazole be used with doxycycline for a total of 14 days of treatment [12]. Sweet recommends broad spectrum antibiotic coverage including coverage of Gram-negative anaerobes for at least 24 hours [11]. Oral therapy and hospital discharge are acceptable when the patient has had a favorable clinical response to therapy. Surgery or drainage should be considered when the patient displays a failure to respond in 48 to 72 hours [9].

Drainage procedures have been increasingly evaluated in the management of TOA. Most recommendations for the management of TOA advise drainage after conservative management has failed [9]. However, several studies show that early drainage of all TOAs is safe and improves outcomes [10, 13] and may be appropriate as a primary therapy [14]. Drainage may be accomplished by CT or ultrasound through the abdomen, vagina, rectum, or transgluteal. Gjelland et al. observed in a retrospective review that, in 302 cases of transvaginal drainage of TOA combined with antibiotics, 93% patients avoided surgery with no major procedure-related complications [10]. Perez-Medina randomized 40 women to traditional conservative management versus antibiotic therapy plus early transvaginal drainage in unruptured TOAs and found that 90% had successful early response versus 65% in the control group (*P*-value ≤  .05) [15]. Moreover, they found that early transvaginal drainage was associated with significantly shorter hospital stays and decreased morbidity. Our study observed only 11 drainage procedures that were split relatively equally between the 2 abscess groups and surgical drainage was not a primary treatment. Thus, we could not assess the effect of drainage on length of hospitalization. 

Regarding antibiotic regimens for TOA, Wiesenfeld and Sweet summarized the response rates of TOAs to medical therapy and showed a 72% success rate in patients treated with clindamycin and aminoglycoside (*n* = 101) versus 82% success rate in patients treated with cefoxitin or cefotetan and doxycycline (*n* = 62) [6]. However, this finding has not been consistently replicated in the literature; another study found this regimen to be less successful [15]. Our data are consistent with the review by Wiesenfeld and Sweet—we found that 9% of patients who were initially treated with cefoxitin and doxycycline required surgical intervention or drainage. These findings should be interpreted with caution, as many of the patients started on the cefoxitin and doxycycline regimen received additional antibiotics.

The strengths of our study are that we have a large number of TOAs despite its being an infrequent outcome. Management of TOA was specifically evaluated with emphasis on the influence of abscess size as a prognostic marker of outcome. The limitations of our study include a relatively small number of large TOAs and the inability to completely control confounding variables such as disease severity. In addition, our study design is unable to completely control confounding factors that may influence the apparent success rate of cefoxitin and doxycycline. Randomized trials are needed to provide sound evidence regarding appropriate antibiotic therapy for TOAs and whether routine drainage of TOAs can decrease prolonged hospitalizations and improve reproductive outcomes. 

## Figures and Tables

**Table 1:** History and sociodemographic characteristics of study population.

*Sociodemographic characteristics*	*N* (%)
Age	
17–19 years	10 (7%)
20–29 years	30 (22%)
30–39 years	51 (38%)
40 years or older	44 (33%)

Race/ethnicity	
White	37 (27%)
Black	92 (68%)
Hispanic/other	6 (4%)

Smoking history	
Yes	64 (47%)
No	45 (33%)
Unknown	20 (19%)

*Reproductive history*	

Gravidity	
0	28 (21%)
1-2	65 (48%)
3 or more	39 (29%)
Missing	3 (2%)

Parity	
0	44 (33%)
1-2	54 (40%)
3 or more	32 (24%)
Missing	5 (4%)

History of GC/CT	38 (28%)

Any contraceptive use	27 (20%)

Condom use	10 (7%)

Tubal ligation	14 (10%)

**Table 2:** Clinical characteristics by presence of TOA.

	TOA	
Clinical Characteristics	Positive *N* (%)	Negative (or not documented) *N* (%)

*Reason presenting for care*		
Pain	127 (94%)	8 (6%)
Median duration of pain	3 days	—
Abnormal bleeding	22 (16%)	113 (84%)
Abnormal discharge	28 (21%)	107 (79%)

*Information at admission*		
Fever	37 (27%)	98 (73%)
Pain	109 (81%)	26 (19%)
Vaginal discharge	42 (31%)	93 (69%)
Rebound	29 (21%)	106 (79%)
Guarding	50 (37%)	85 (63%)
Uterine tenderness	36 (27%)	99 (73%)
Adnexal tenderness	69 (51%)	66 (49%)
Cervical motion tenderness	65 (48%)	70 (52%)
Cervical culture	91 (67%)	44 (33%)
GC (*n* = 91)	11 (12%)	77 (85%)
CT (*n* = 91)	13 (14%)	74 (81%)
CT performed	106 (79%)	29 (21%)
US performed	94 (70%)	41 (30%)
MRI performed	3 (2%)	132 (98%)
WBC, mean (SD)**	14.2 (5.9)	—
WBC		
<10	37 (27%)	—
10 to <15	46 (34%)	—
15+	52 (39%)	—
Unilateral abscess^†^	89 (66%)	—
Largest abscess size (cm), *mean *(SD) (Note: range = 1.5–16 cm)	6.7 (2.7)	—
Abscess size		
0 to 4 cm	43 (32%)	—
5 to 8 cm	69 (51%)	—
>8 cm	23 (17%)	—

*87 patients (64%) had no information on HIV in medical record

**WBC not documented for 5 patients

^†^Information missing for 21 patients.

**Table 3:** Outcomes and initial antibiotic regimen by abscess size.

	All patients (*N* = 135)	Abscess ≤8 cm (*N* = 112)	Abscess >8 cm (*N* = 23)	*P*-value
# days hospitalized mean (SD)	4.6 (3.6)	4.4 (3.4)	5.7 (4.2)	.09

# days febrile (>38.2)	1.1 (1.8)	1.10 (1.6)	1.35 (2.5)	.65

*N* (%)				

Requiring drainage/surgery	42 (31%)	32 (29%)	10 (43%)	.16
Drainage*	11 (8%)	9 (8%)	2 (9%)	.31
Surgery	34 (25%)	26 (23%)	8 (35%)	.24

Number of antibiotics used, mean (SD)	2.8 (1.2)	2.7 (1.2)	3.1 (1.3)	.20

Antibiotic regimen*				
Cefoxitin and doxycycline	57 (43%)	50 (45%)	7 (30%)	.14
Ciprofloxacin and Flagyl	11 (8%)	7 (6%)	4 (17%)	
Gentamicin and clindamycin	18 (14%)	13 (12%)	5 (22%)	
Other	46 (35%)	39 (35%)	7 (30%)	

Any complication	18 (13%)	10 (9%)	8 (35%)	<.01

*P-value estimated from Fisher's exact test.

**Table 4:** Outcomes of surgery and drainage by initial antibiotic regimen.

	No surgery/ drainage	Surgery/ drainage	*P*-value
Cefoxitin/Doxycycline	52 (91%)	5 (9%)	**<.0001**
Ciprofloxacin/Flagyl	5 (45%)	5 (55%)	
Gentamicin/Clindamycin	9 (50%)	9 (50%)	
Other	27 (59%)	19 (41%)	
